# Pulmonary metastasis secondary to abiraterone‐resistant prostate cancer with homozygous deletions of BRCA2: First Japanese case

**DOI:** 10.1002/iju5.12224

**Published:** 2020-10-15

**Authors:** Mizuki Izawa, Takeo Kosaka, Kohei Nakamura, Junna Oba, Tomoyuki Hishida, Hiroshi Hongo, Shuji Mikami, Hiroshi Nishihara, Mototsugu Oya

**Affiliations:** ^1^ Department of Urology Keio University School of Medicine Tokyo Japan; ^2^ Genomics Unit Keio Cancer Center Keio University School of Medicine Tokyo Japan; ^3^ Division of Thoracic Surgery Department of Surgery Keio University School of Medicine Tokyo Japan; ^4^ Division of Diagnostic Pathology Keio University Hospital Tokyo Japan

**Keywords:** abiraterone acetate, BRCA2, DNA damage repair genes, next‐generation sequencing, prostate cancer

## Abstract

**Introduction:**

Most metastatic prostate cancers acquire the capacity for androgen‐independent growth and become resistant to androgen deprivation therapy. A patient‐focused treatment strategy is needed for aggressive castration‐resistant prostate cancer.

**Case presentation:**

We report the case of a 62‐year‐old man who presented with prostatic adenocarcinoma who was treated by radiation and combined androgen blockade. After completion of first‐line therapy, he was diagnosed with multiple metastatic castration‐resistant prostate cancer in the lung. Second‐line therapy with abiraterone acetate resulted in partial remission of the lung metastases. Thoracic surgery was performed to remove the single lung metastasis remaining. Next‐generation sequencing of the specimens demonstrated homozygous loss of *BRCA2*. We note in this case a heterogeneous response to abiraterone acetate may be related to the somatic *BRCA2* deletions.

**Conclusions:**

We present the first Japanese case of a metastatic abiraterone acetate‐resistant castration‐resistant prostate cancer accompanied by BRCA2 mutation.

**Abbreviations & Acronyms**
ABI: abiraterone acetate
AR: androgen receptor
CRPC: castration‐resistant PCa
CT: computed tomography
DDR: DNA damage response
DSB: double‐strand break
IMRT: intensity‐modulated radiation therapy
MRI: magnetic resonance imaging
NGS: next‐generation sequencing
PARP: poly (ADP‐ribose) polymerase
PCa: prostate cancer
PSA: prostate‐specific antigen
VATS: video‐assisted thoracic surgery
VUS: variant of unknown significance

**Keynote message**

We present the first Japanese case of a 62‐year‐old male with metastatic ABI‐resistant CRPC accompanied by BRCA2 mutation. This case report indicates that the ongoing examination of gene mutations may have a significant impact on future treatments for PCa.

## Introduction

PCa is the second‐leading cause of the cancer deaths among men in Western countries.^1^ The incidence of PCa is still on the rise in Japan.^2^ Most metastatic PCa become both resistant to both castration and androgen deprivation therapy.^3^ None of CRPC therapeutic modalities are curative and acquired resistance is inevitable. Precision medicine and a more individual patient focused treatment strategy might be facilitated by the use of NGS.

Genetic aberrations may be directly involved in the development, invasiveness, and metastatic potential of a given cancer. Recently, it has become clear that genetic aberrations involving *BRCA1* and *BRCA2* genes are heavily involved in the acquisition of resistance to treatment in PCa.^4^

We present here the first Japanese case of PCa that was resistant to second‐generation antiandrogens and demonstrated homozygous loss of *BRCA2*.

## Case presentation

**Fig. 1:**
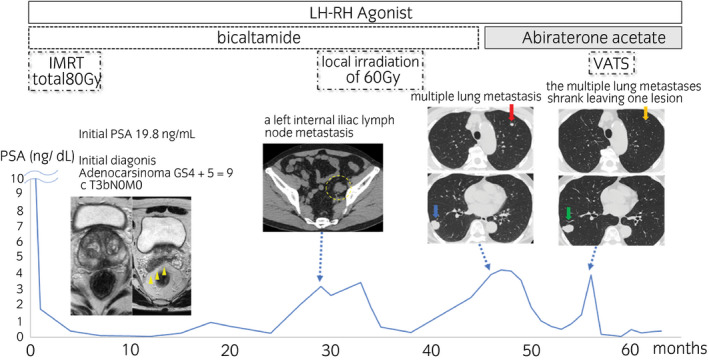
Serum PSA level and time course for treatment with CT and MRI as indicated. MRI revealed a prostate tumor invading the seminal vesicles; cT3b (arrowheads). CT scan 29 months after initial androgen deprivation therapy revealed a left internal iliac lymph node metastasis (dotted circle). Multiple lung metastases disappeared in response to treatment with ABI; one lesion persisted at 9 months (the areas indicated by the red and blue arrows changed, as the areas indicated by the yellow and green arrows, respectively).

A 62‐year‐old man presented for evaluation of an elevated serum PSA (19.8 ng/mL). Needle biopsy led to the diagnosis of prostatic adenocarcinoma with a Gleason score of 4 + 5 = 9. MRI was notable for prostate tumor invading the seminal vesicles, although skeletal scintigraphy revealed no bone metastasis (Fig. 1) and no visceral metastases were detected on abdominal CT. Based on these results, the clinical stage was determined to be cT3bN0M0. First‐line treatment included IMRT (total 80 Gy) with combined androgen blockade. Serum PSA decreased to 0.05 ng/mL during the treatment but increased to 2.64 ng/mL at 29 months later. CT scan at that time revealed a metastatic left internal iliac lymph node. The patient was diagnosed with CRPC and treated with local irradiation of 60 Gy to the lymph node. After radiation, serum PSA dropped to its nadir at 0.30 ng/mL. Eight months later, his serum PSA level increased to 4.25 ng/mL in association with multiple lung metastases detected on CT (Fig. 1). The patient was treated with ABI. Nine months after initiating ABI therapy, most of the lung metastases had disappeared, although one lesion remained. This ABI‐resistant solitary lung nodule was diagnosed as an oligometastatic lesion; this definition implies that an appropriate surgical procedure may be curative. To remove the remaining lung metastasis, VATS was performed. After the procedure, the PSA level again dropped to 0.04 ng/mL; serum PSA has remained <1.0 ng/mL for 10 months (Fig. 1).

**Fig. 2:**
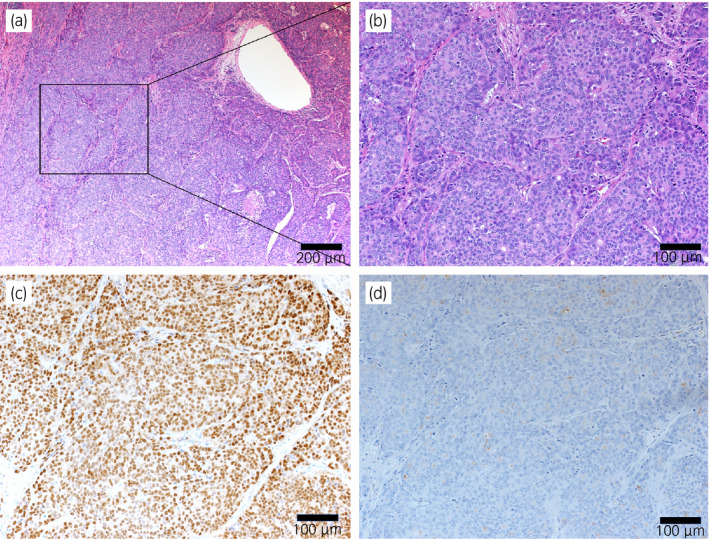
(a) Low power view of hematoxylin and eosin (H&E) stained lung tumor tissue. (b) High power view of the H&E staining of tissue (a) showed morphological features compatible with metastatic PCa. Immunostaining of tissue to detect (c) AR and (d) PSA. The scale bar indicates 200 μm in (a), 100 μm in others.

The lung tumor cells proliferated in solid pattern that was consistent with the diagnosis of metastatic PCa (Fig. 2a,b). Immunohistochemically, tumor cells were strongly positive for AR, weakly positive for PSA (Fig. 2c,d) and negative for both thyroid transcription factor 1 and p40 which are markers for adenocarcinoma, and squamous cell carcinoma of the lung, respectively.

**Fig. 3:**
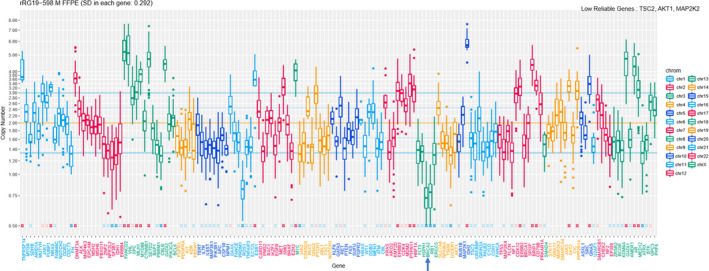
Genomic profiling of cancer‐related genes. The horizontal axis indicates the genes examined and the vertical axis indicates the copy number. The blue arrow points to BRCA2.

We performed a genomic analysis of this resected metastatic tumor using the NGS clinical sequencing system at our hospital (Table S1).^5^, ^6^ We also identified homozygous (biallelic) deletions of *BRCA2* and *RB1* in the tumor tissue (Fig. 3). *CDK12* somatic point mutation (p.Q937*) was detected as a pathogenic variant. All variants detected, including VUS are presented in Table 1.

**Table 1:** Detailed information about gene alterations

Gene alterations	Variant allele frequency (%)	Pathogenicity
*CDK12* Q937*	72.2	Pathogenic
*CDK12 R1008Q*	25.5	VUS
*IL6ST V771A*	42.1	VUS
*AMER1*	G447S	VUS

Oncogene targeted amplification resulted in the detection of *IDH2*, *AR*, *MYC*, *ERBB2*, *ERBB3*, *ESR1*, *NRAS*, *SMO*, and *AKT2*. All relevant copy numbers are presented in Table 2. Tumor mutation burdens were calculated at 4.0 single nucleotide variants per million bp. Copy number variation and variant allele frequency plots (Fig. 3) indicated a high frequency of loss of heterozygosity and scattered allelic imbalance; these findings are detected comparatively frequently in homologous recombination‐deficient tumors.

**Table 2:** Detailed information about gene copy number alterations

Genes	Copy number	
*BRCA2*	0	Homozygous deletion
*RB1*	0	Homozygous deletion
*IDH2*	14.3	Amplification
*AR*	9.6	Amplification
*MYC*	8.9	Amplification
*ERBB2*	6.2	Amplification
*ERBB3*	5.6	Amplification
*ESR1*	7.3	Amplification
*NRAS*	6.1	Amplification
*SMO*	6.1	Amplification
*AKT2*	6.3	Amplification

## Discussion

The mechanism by which PCa acquires treatment resistance is closely linked to genetic abnormalities of DDR signaling pathways including *BRCA1*/*2* loss. *BRCA2* performs homologous recombination‐mediated DNA repair and provide critical contributions for the repair of DSBs in genomic DNA. Mutations in these genes lead to the inactivation of homologous DNA repair mechanisms and promote the development of DSBs. *BRCA1/2* has been identified as critical therapeutic target for PCa. In recent years, *BRCA2* aberrancies have been identified in 12% of metastatic CRPC cases.^7^

The loss of function mutations or deletions in *BRCA2* has been reported to be associated with the inactivation of homologous DNA repair mechanisms and with the development of double‐stranded DNA breaks, rendering these cells more susceptible to PARP inhibitors. Olaparib is a PARP inhibitor that has just been approved for patients with PCa and DDR mutations including *BRCA2* loss.^8^, ^9^ In the future, PARP inhibitors may be the optimal treatment for PCa with *BRCA2* gene alteration in Japan.^10^, ^11^

There are various opinions concerning the relationship between *BRCA* alteration and responsiveness for treatment using ABI or enzalutamide. Annala *et al*.^12^ reported that patients with *BRCA2* germline mutation exhibited poor responses to therapies that targeted the AR signaling axis, and in contrast, Antonarakis *et al*.^13^ reported a good response to AR targeting therapies. Mateo *et al*.^14^ have recently published a new analysis that included somatic *BRCA2* alteration in addition to germline mutations; these results suggest that *BRCA2* alterations are powerful predictors of resistance to AR targeting therapies; these latest data include somatic alterations of DDR‐related genes. However, these reports were established in Western patient cohorts; the genomic and biological implications of these findings with respect to Japanese patients remain unclear. In our case, we observed a heterogeneous response to ABI; one lung metastasis remained after treatment. The limitation is that gene profiling of patient’s peripheral blood and initial prostate biopsy specimen were not performed. However, homozygous deletion in *BRCA2* gene on a wide range of 13q was identified. If the 13q deletion occurred as a germline event, it would cause malformations that affect various organ systems. There were no such findings in this case; *BRCA2* homozygous deletion was thought to have occurred as a somatic event.

Recently, we have reported several cases of somatic and germline mutations in DDR‐related genes.^6^, ^11^ Collectively, these trials and case reports indicate that the ongoing examination of gene mutations may have a significant impact on future treatments for PCa in addition to the use of PARP inhibitors or platinum‐based chemotherapy. Nonetheless, because PCa tends to be heterogeneous within each subtype, genetic testing should be used to develop more specific treatment strategies.

## Conclusions

We present the first Japanese case of metastatic ABI‐resistant CRPC accompanied by *BRCA2* homozygous deletion.

## Conflict of interest

The authors declare no conflict of interest.

## Supporting information


**Table S1**. 160 genes examined in the PleSSision‐Rapid test, which is used for all genome sequencing‐related analyses in Keio University Hospital.

## References

[1] Siegel RL , Miller KD , Jemal A . Cancer statistics, 2020. CA Cancer. J. Clin. 2020; 70: 7–30. 31912902 10.3322/caac.21590

[2] Saito E , Hori M , Matsuda T , Yoneoka D , Ito Y , Katanoda K . Long‐term trends in prostate cancer incidence by stage at diagnosis in Japan using the multiple imputation approach, 1993–2014. Cancer Epidemiol. Biomarkers Prev. 2020; 29: 1222–8. 32169995 10.1158/1055-9965.EPI-19-1228

[3] Fujimoto N . Role of the androgen‐androgen receptor axis in the treatment resistance of advanced prostate cancer: From androgen‐dependent to castration resistant and further. J. UOEH. 2016; 38: 129–38. 27302726 10.7888/juoeh.38.129

[4] Patel VL , Busch EL , Friebel TM *et al* Association of Genomic Domains in BRCA1 and BRCA2 with prostate cancer risk and aggressiveness. Cancer Res. 2020; 80: 624–38. 31723001 10.1158/0008-5472.CAN-19-1840 PMC7553241

[5] Hayashi H , Tanishima S , Fujii K *et al* Genomic testing for pancreatic cancer in clinical practice as real‐world evidence. Pancreatology 2018; 18: 647–54. 30055942 10.1016/j.pan.2018.07.006

[6] Watanabe K , Kosaka T , Aimono E *et al* Japanese case of enzalutamide‐resistant prostate cancer harboring a SPOP mutation with scattered allelic imbalance: response to platinum‐based therapy. Clin. Genitourin. Cancer 2019; 17: e897–e902. 31296452 10.1016/j.clgc.2019.06.005

[7] Abida W , Armenia J , Gopalan A *et al* Prospective genomic profiling of prostate cancer across disease states reveals germline and somatic alterations that may affect clinical decision making. JCO Precision Oncol. 2017; 1–16. 10.1200/PO.17.00029 PMC5558263 28825054

[8] Martin GA , Chen AH , Parikh K . A novel use of Olaparib for the treatment of metastatic castration‐recurrent prostate cancer. Pharmacotherapy 2017; 37: 1406–14. 28895177 10.1002/phar.2027

[9] de Bono J , Mateo J , Fizazi K *et al* Olaparib for metastatic castration‐resistant prostate cancer. N. Engl. J. Med. 2020; 382: 2091–102. 32343890 10.1056/NEJMoa1911440

[10] Kosaka T , Hongo H , Aimono E *et al* A first Japanese case of neuroendocrine prostate cancer accompanied by lung and brain metastasis with somatic and germline BRCA2 mutation. Pathol Int. 2019; 69: 715–20. 31631483 10.1111/pin.12860 PMC6972566

[11] Hongo H , Kosaka T , Aimono E , Nishihara H , Oya M . Aggressive prostate cancer with somatic loss of the homologous recombination repair gene FANCA: a case report. Diagn. Pathol. 2020; 15: 5. 31931827 10.1186/s13000-019-0916-z PMC6958728

[12] Annala M , Struss WJ , Warner EW *et al* Treatment outcomes and tumor loss of heterozygosity in germline DNA repair‐deficient prostate cancer. Eur. Urol. 2017; 72: 34–42. 28259476 10.1016/j.eururo.2017.02.023

[13] Antonarakis ES , Lu C , Luber B *et al* Germline DNA‐repair gene mutations and outcomes in men with metastatic castration‐resistant prostate cancer receiving first‐line abiraterone and enzalutamide. Eur. Urol. 2018; 74: 218–25. 29439820 10.1016/j.eururo.2018.01.035 PMC6045965

[14] Mateo J , Cheng HH , Beltran H *et al* Clinical outcome of prostate cancer patients with germline DNA Repair mutations: Retrospective analysis from an International Study. Eur. Urol. 2018; 73: 687–93. 29429804 10.1016/j.eururo.2018.01.010 PMC6745088

