# Supplementary material for: Pulmonary metastasis secondary to abiraterone‐resistant prostate cancer with homozygous deletions of BRCA2: First Japanese case
Source: IJU Case Rep. 2020 Oct 15;4(1):14–7. doi: 10.1002/iju5.12224 (PMC7784740; doi:10.1002/iju5.12224)
Supplement: Supplementary file 1 — Table S1. 160 genes examined in the PleSSision‐Rapid test, which is used for all genome sequencing‐related analyses in Keio University Hospital. [file IJU5-4-14-s001.docx]

Supplemental Table； 160 genes examined in the PleSSision-Rapid test

| ABL1 | AKT1 | AKT2 | ALK | AMER1 | APC | AR | ARID1A |
| --- | --- | --- | --- | --- | --- | --- | --- |
| ARID2 | ASXL1 | ATM | ATRX | BAP1 | BCL6 | BCOR | BRAF |
| BRCA1 | BRCA2 | BRIP1 | BTK | BUB1B | CARD11 | CBL | CBLB |
| CD79A | CD79B | CDC73 | CDH1 | CDK12 | CDK4 | CDKN2A | CHEK2 |
| CIC | CREBBP | CRLF2 | CSF1R | CTNNB1 | CYLD | DAXX | DDB2 |
| DDR2 | DICER1 | DNMT3A | ECT2L | EGFR | EP300 | EPCAM | ERBB2 |
| ERBB3 | ERBB4 | ERCC5 | ESR1 | EZH2 | FAM46C | FANCA | FANCD2 |
| FANCE | FAS | FBXO11 | FBXW7 | FGFR2 | FGFR3 | FH | FLCN |
| FLT3 | FUBP1 | GATA1 | GATA2 | GATA3 | GNA11 | GNAQ | GNAS |
| GPC3 | GRIN2A | H3F3A | HIST1H3B | HNF1A | HRAS | HSPH1 | IDH1 |
| IDH2 | IKZF1 | IL6ST | IL7R | JAK1 | JAK2 | JAK3 | KDM6A |
| KDR | KIT | KLF6 | KMT2D | KRAS | MAP2K1 | MAP2K2 | MAP2K4 |
| MAP3K1 | MAP4K3 | MDM2 | MED12 | MEN1 | MET | MLH1 | MSH2 |
| MSH6 | MTOR | MUTYH | MYC | MYD88 | NF1 | NF2 | NFE2L2 |
| NFKBIA | NOTCH1 | NOTCH2 | NPM1 | NRAS | PALB2 | PAX5 | PBRM1 |
| PDGFRA | PHF6 | PIK3CA | PIK3R1 | PMS2 | PPP2R1A | PRDM1 | PRKAR1A |
| PTCH1 | PTEN | PTPN11 | RAC1 | RB1 | RET | ROS1 | SDHB |
| SETD2 | SF3B1 | SLC7A8 | SMAD4 | SMARCA4 | SMARCB1 | SMO | SPOP |
| SRC | STK11 | SUFU | TERT | TNFAIP3 | TNFRSF14 | TP53 | TSC1 |
| TSC2 | TSHR | U2AF1 | VHL | WT1 | XPC | ZNF2 | ZRSR2 |

We evaluated the frequency of mutations in 160 cancer-related genes in genomic material isolated from the metastatic nodule in the lung tumor. Sections (10 µm) were dissected to provide > 20% tumor cells in the specimens and to minimize the incidence of necrosis. Genomic testing was performed on a PleSSision-Rapid internal clinical sequencing apparatus, which is used for all genome sequencing-related analyses in our hospital (Keio University Hospital).
